# Intracellular and Extracellular Roles of Granzyme K

**DOI:** 10.3389/fimmu.2021.677707

**Published:** 2021-05-04

**Authors:** Annemieke C. Bouwman, Kim R. van Daalen, Sandra Crnko, Toine ten Broeke, Niels Bovenschen

**Affiliations:** ^1^ Department of Pathology, University Medical Center Utrecht, Utrecht, Netherlands; ^2^ Cardiovascular Epidemiology Unit, Department of Public Health & Primary Care, University of Cambridge, Cambridge, United Kingdom; ^3^ Center for Translational Immunology, University Medical Center Utrecht, Utrecht, Netherlands

**Keywords:** cytotoxic cells, granzyme K, cytotoxicity, inflammation, disease

## Abstract

Granzymes are a family of serine proteases stored in granules inside cytotoxic cells of the immune system. Granzyme K (GrK) has been only limitedly characterized and knowledge on its molecular functions is emerging. Traditionally GrK is described as a granule-secreted, pro-apoptotic serine protease. However, accumulating evidence is redefining the functions of GrK by the discovery of novel intracellular (e.g. cytotoxicity, inhibition of viral replication) and extracellular roles (e.g. endothelial activation and modulation of a pro-inflammatory immune cytokine response). Moreover, elevated GrK levels are associated with disease, including viral and bacterial infections, airway inflammation and thermal injury. This review aims to summarize and discuss the current knowledge of i) intracellular and extracellular GrK activity, ii) cytotoxic and non-cytotoxic GrK functioning, iii) the role of GrK in disease, and iv) GrK as a potential therapeutic target.

## Introduction

Granzymes are a family of serine proteases traditionally known for their role in promoting cytotoxicity of foreign, infected or neoplastic cells. Granzymes induce (apoptotic) cell death mediated by a collective of cytotoxic lymphocytes (CLs) (e.g. cytotoxic T lymphocytes (CTLs), natural killer (NK) cells). There are five human granzymes (granzyme A (GrA), GrB, GrH, GrK and GrM) currently identified, whereas mice have ten known granzymes (GrA-G, GrK, GrM and GrN) (1). Whilst human granzymes are homologous in amino acid sequence (40%), they vary in their primary substrate specificity, function(s) and are uniquely expressed in distinct cell types (2, 3). Upon activation, CTLs and NK cells induce apoptosis *via* the extrinsic death-receptor pathway or the granule secretory pathway involving the pore-forming protein perforin and granzymes (4). The latter are collectively stored in intracellular granules and delivered by CLs to the immunological synapse after recognition of a target cell (4). Consecutively, aided by perforin, granzymes are released in the cytosol of target cells, where they cleave intracellular substrates and activate signaling pathways (4).

Although granzymes are traditionally described as primarily being involved in immune-targeted cell death, emerging clinical and biochemical evidence suggests additional roles for granzymes (5, 6). For example, GrB acts in autoimmune diseases by directly cleaving or aiding in the production of autoantigens (7). Additionally, various granzymes (GrA, GrB, GrM) are reported to induce inflammation and interfere in viral replication (3, 8–10) and all human granzymes target hnRNP K which reduces tumour cell viability (11). Apart from intracellular activity, granzymes exist in the extracellular space, thereby having the potential to exert effects on both sides of the cellular membrane. Illustratively, GrA, which shares homology and enzymatic activity with GrK, and GrB can degrade extracellular matrix (ECM) possibly leading to cell death through anoikis (12). Extracellular granzymes are particularly upregulated in diseased individuals (e.g. in skin inflammation, viral and bacterial infections) (9, 13–16). Combined, this supports the current view that granzymes possess extracellular roles in addition to their traditionally described intracellular roles. Similarly, it has been suggested that GrK has non-cytotoxic roles, including the augment of GrA-induced pro-inflammatory processes (17, 18).

In comparison to GrA (a tryptase) and GrB (an asp-ase), little is known about the molecular functioning of the only other tryptase in the granzyme family; GrK (19). Human GrK was first discovered in 1988 after purification from human peripheral blood mononuclear cells (20). GrK is expressed by CTLs, natural killer T cells (NKT), γδ T cells and CD56^bright+^ NK cells (21–23). Similar to its closest homolog GrA, GrK displays tryptase-like activity cleaving substrates after basic Arg or Lys (24). Since GrA and GrK are both tryptases and have some substrates in common, GrK has long been viewed as a redundant enzyme for GrA. However, this concept is now debated due to the unique substrates and functionality of GrK (25). The GrK gene (GZMK) is closely linked to GZMA on chromosome 5, likely originating from gene duplication (1, 26). Human GrK is synthesized as inactive pre-pro-granzyme (zymogen) containing a signal dipeptide directing pre-pro-GrK to the endoplasmic reticulum (ER) (27). Removal of the dipeptide [e.g. by granular cathepsin (28)] results in conformational change and subsequent catalytic activation (29). Mature GrK exists as a monomer with four disulfide bridges and no free cysteine (Cys) residues. The three-dimensional structure highly relates to trypsin and other related granzymes (27). GrK contains a heparin-binding site, an activation domain with proteolytic activity and a nonspecific substrate-binding template strand (24, 30). Various GrK substrates have been reported, including nucleosome assembly protein SET, heterogeneous nuclear ribonucleoprotein (hnRNP) K, β-tubulin and α-tubulin (25, 31). Inter-alpha inhibitor 1 (IαIp), which circulates in the plasma of healthy individuals, is a physiological GrK inhibitor (32). Similar to the other granzymes, the traditional role of GrK is debated and (extracellular) functions of GrK in promoting inflammation and infections are emerging.

Both intra- and extracellular GrK target physiological substrates (**Table 1**), dependent or independent on its catalytic activity. Furthermore, GrK is released in plasma of patients suffering from e.g. autoinflammatory diseases, suggesting a potential role in the biological impact of the diseases. Yet, little has been described on the potential non-cytotoxic and extracellular functioning of GrK thus far. Therefore, this review aims to comprehensively describe the diverse roles of Granzyme K and its potential as therapeutic target.

**Table 1:** Intracellular and extracellular substrates of GrK and suggested biological impact.

Substrate	GrA substrate?	(Extra) cellular location	(Suggested) biological impact	Reference
*Intracellular substrates*				
SET complex	+^(89)^	Nucleus	NM23H1-induced DNA nicks, chromatin condensation and apoptotic morphology.	(25, 33)
Bid	-^(89)^	Mitochondria	Disruption of the outer mitochondrial membrane and release of cytochrome c and endonuclease G.	(34)
Ape1	+^(89)^	Nucleus	Inhibits its redox activity facilitating intracellular ROS accumulation and enhancing GrK-induced cell death.	(35)
VCP		ER	Inhibition of ERAD components and initiation of ER stress leading to ROS accumulation and cytotoxicity.	(36)
p53		Nucleus, mitochondria	Cleavage products p13, p35 and p40 induce transcription of p21, PG13, MDM and mitochondrial disruption, leading to ROS accumulation and cytotoxicity.	(37)
Importin 1α/β		Nucleus	Inhibition of viral replication by preventing NP/viral RNA complex formation.	(38)
β-tubulin		Cytoskeleton	Potential novel cell death pathway and terminating viral production in infected cells during NK cell attack.	(25)
hnRNP K	+^(12)^	Nucleus	Potential novel cell death pathway and/or terminating viral production in infected cells during NK cell attack.	(25)
*Extracellular substrates*				
PAR-1		Cell membrane	Activation of PAR-1 mediating endothelial activation and release of pro-inflammatory cytokines.	(39, 40)
LPS	-^(90)^	ECM	Removal of LPS molecules from micelles and transfer to CD14 and TLR4, promoting cytokine expression.	(41)

Bid, BH3 interacting-domain death agonist; Ape1, Apurine/apyrimidine endonuclease 1; VCP, Vasolin-containing protein; ER, Endoplasmic Reticulum; ROS, reactive oxygen species; ERAD, ER associated protein degradation; ECM, Extracellular matrix; NK cell, Natural Killer cell; NP, Nuclear protein; LPS, Lipopolysaccharides; PAR-1, Proteinase-activated receptor 1.

## Intracellular GrK Activity

### Cytotoxicity

**Figure 1:**
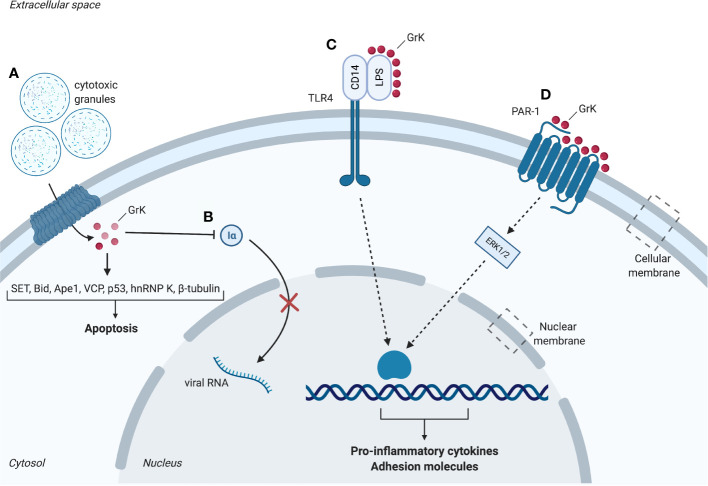
Intracellular and extracellular substrates of granzyme K (GrK). **(A)** Cytotoxic granules containing perforin and granzymes enter the cell. GrK binds or cleaves substrates SET, Bid, Ape1, VCP, p53, hnRNP K and β-tubulin which promote apoptosis. **(B)** GrK inhibits replication of influenza A through cleavage of importin-1α and -β, thereby hindering nuclear uptake of importin-1α and -β. **(C)** GrK cleaves and activates PAR-1. Downstream phosphorylation of ERK1/2 results in a pro-inflammatory cytokine response (e.g. IL- 1β, MCP-1, IL-6, IL-8) and increased expression of adhesion molecules (e.g. ICAM-1, VCAM-1, E-selectin). **(D)** GrK facilitates binding between LPS and CD14. CD14 binds to TLR4 which leads to a pro-inflammatory cytokine response. Iα, importin-1α; LPS, lipopolysaccharides; PAR-1, Protease-activated receptor 1; TLR4, Toll-like receptor 4. Red spheres: granzyme K. Figure created with Biorender.com.

Pioneering research on rat GrK initially classified GrK as a DNA-fragmenting protease and identified it as fragmentin. This was a result of the observation that GrK induced YAC-1-derived DNA to be cleaved in oligonucleosome-sized fragments resulting in the formation of severe chromatin condensation (42). The first *in vitro* study on the apoptotic capacity of human GrK combined with perforin suggested that it induces non-apoptotic cell death *via* provoking mitochondrial dysfunctioning and generation of reactive oxygen species (ROS) (43). Further *in vitro* studies showed that cleavage of several intracellular GrK substrates results in cytotoxicity (**Figure 1**), including proteins of the SET complex, BH3 interacting-domain death antagonist (Bid), vasolin-containing protein (VCP) and p53.

Early studies on human GrK showed that GrK activates caspase-independent apoptosis by cleaving the nucleosome assembly protein (NAP) SET in its recombinant and native form or in intact cells *in vitro* (33). This results in the disruption of SET and loss of inhibition of GrK-activated DNase (GAAD) functioning. Consequently, the GAAD NM23H1 nicks chromosomal DNA, resulting in chromatin condensation and apoptotic nuclear morphology comparable to GrA (33). GrK also targets other SET complex proteins including DNA-binding protein HMG2 and redox factor-1/apurinic apyrimidinic endonuclease 1 (Ape1), an endonuclease antagonizing ROS generation (33, 35). The initial suggestion that GrK and GrA show redundant specificity and function is supported by the observation that both granzymes cleave SET, Ape1 and HMG2 with similar degradation fragments (**Table 1**) (33). As a physiological substrate of GrK, Ape1 cleavage facilitates intracellular accumulation of ROS. This may be the first step in GrK-mediated cell death as ROS accumulation takes part in a positive feedback loop wherein disruption of mitochondria leads to additional ROS release, also known as ROS-induced ROS release (44). GrK reportedly induces ROS accumulation *in vitro*, supporting this hypothesis (34, 43, 44).

Other potential pathways triggering GrK-induced apoptosis depend on mitochondrial damage and ER stress, both resulting in ROS accumulation (36, 37). Like GrB, human GrK is shown to degrade recombinant and native Bid to truncated Bid (tBid) *in vitro*, enabling it to disrupt the outer mitochondrial membrane (34). This leads to release of pro-apoptotic cytochrome c and endonuclease G, suggesting GrK caspase-mediated apoptosis. These results are in contrast with earlier findings suggesting rat GrK does not trigger cytochrome c release *in vitro* (45). This may be explained due to granzyme orthologues not completely sharing substrate specificity (46). Furthermore, VCP is an important component of the endoplasmic-reticulum-associated protein degradation (ERAD) pathway that eliminates misfolded proteins and has ATPase activity. *In vitro* GrK can bind and cleave VCP, as well as other ERAD components Ufd1 and Np14 (36). The resulting inhibition of ERAD leads to the accumulation of misfolded proteins and ER stress (36). Adaptive responses to limit ER stress include nutrient starvation and transcriptional activation of RNases which degrade misfolded proteins (47). When the adaptive response is not sufficient to overcome ER stress, the cell will undergo apoptosis (47). ER stress, as well as tBid-mediated disruption of the outer mitochondrial membrane, may lead to additional accumulation of ROS (34). Lastly, p53 is a physiological target of GrK, processing p53 to three pro-apoptotic fragments: p13, p35 and p40 (37). Once cleaved into their active form, these products cause mitochondrial disruption and upregulation of p21, PG13 and MDM2 transcription (37). Intracellular GrK-challenged tumor cells are killed in a p53-dependent manner (37).

These results identify various intracellular GrK substrates *in vitro*, including SET, Bid, Ape1, VCP and p53 leading to cytotoxicity in caspase-independent ways through DNA damage, mitochondrial damage and ROS accumulation (**Figure 1**). Importantly, next to sharing some substrates with GrA (**Table 1**), GrK has thus shown to possess its own unique functions cleaving specific substrates in addition to being a functional GrA back-up.

### Non-Cytotoxic Roles

In contrast to *in vitro* studies on human and rat GrK (42), *in vitro* mice studies report no cytotoxicity of mouse GrK (mGrK) (17). Analyzing the same markers of apoptosis as in previous studies (phosphatidylserine externalization, mitochondrial membrane integrity and ROS accumulation), mGrK concentrations up to 600 nM (with delivery agent) and 1200 nM (without delivery agent) did not induce apoptosis in mouse embryonic fibroblasts and EL4 cells (17). Similar results were obtained *in vivo* using GrK-deficient (GrK^-/-^) mice (48). When comparing the apoptotic potential of GrK^-/-^ mice with WT mice, no essential role in cytotoxicity for GrK was found (48). Hence, the apoptotic potential of GrK has remained controversial (48).

This controversy has also been reported for GrA. Despite early studies demonstrating GrA induces cell death *in vitro* (49, 50), others identified GrA as a pro-inflammatory granzyme, unable to induce apoptosis *in vivo* in mice and humans (51). For GrB, nanomolar concentrations were sufficient to induce apoptosis in Jurkat cell-free extracts, suggesting high cytotoxicity (52). In studies proposing GrA’s and GrK’s cytotoxic activity however, micromolar concentrations are required, suggesting lower cytotoxicity than GrB (53). Whilst granzymes are conserved in various organisms, differences in granzyme substrate specificity and function among species may explain this conflicting data. Further *in vivo* studies on human GrK using physiological GrK levels are needed to establish its cytotoxic potential.

GrK also targets intracellular non-cytotoxic substrates such as importin α1 or β *in vitro*, destabilizing their association to generate the ternary import complex for transportation of cytoplasmic cargos (α1/β dimer) (38) (**Figure 1**). Proteomic studies furthermore identified intracellular substrates β-tubulin, the microtubule network protein, and the pre-mRNA-binding protein hnRNP K (25) (**Table 1**). Both proteins play important roles in cellular physiology making them relevant for cell survival. Cleavage of β-tubulin may impair tubulin polymerization, whereas cleavage of hnRNP K may inhibit or rescue the translation of proteins involved in cell death. Cleavage of these proteins raised the potential of a new cell death pathway induced by GrK different from GrA (25). hnRNP K downregulation was shown to promote a mixture of exon inclusion and exon skipping events affecting various apoptotic proteins (54). However, thus far, the physiological role of β-tubulin and hnRNP K cleavage remains unclear (25).

GrK is suggested to augment GrA-induced pro-inflammatory processes by cleaving the same substrates differentially - based on the high display of GrK substrate specificity (17) (**Table 1**). This may be through stimulation of target cells to secrete pro-inflammatory cytokines. mGrK induces pro-inflammatory interleukin-1β (IL-1β) maturation and secretion in LPS-sensitized peritoneal macrophages (PEMØ) *in vitro* (17). In this research, it was not established whether this effect was dependent on intra- or extracellular modulation by mGrK. However, nanomolar, physiological concentrations of mGrK in combination of perforin were sufficient to induce IL-1β release (>30 nM), whereas only high, non-physiological concentrations of mGrK alone induced IL-1β release (600 and 1000 nM) (17). Hence, mGrK may induce IL-1β release of primed PEMØs dependent on intracellular modulation. To date, no additional intracellular substrates of GrK have been described. All in all, several non-cytotoxic intracellular targets and functions have been described for GrK. These include importin α1 or β, β-tubulin and hnRNP K.

## Extracellular GrK Activity

GrK circulates in the extracellular space in different forms. In healthy individuals, GrK can form complexes ranging from 150-250 kDa (inactive multimer), whereas its free form is 26 kDa (monomer) (14). Multiple mechanisms have been suggested to result in extracellular GrK release. These include i) escape from the immunological synapse, ii) granzyme release post degranulation, iii) degranulation induced by chemokines or iv) cytokines and v) granzyme release following integrin-ECM proteins interaction (55). Extracellular GrK, when administered in the absence of a delivery agent such as perforin, is not considered cytotoxic *in vitro* (39, 40, 56). Furthermore, GrK, among other cell types, is expressed by CD56^bright+^ NK cells, and classically activated macrophages, which either express none or negligible levels of perforin (21, 56). The presence of GrK in the extracellular space and its synthesis in the absence of perforin suggests GrK has additional functions in addition to its classically described perforin-mediated intracellular activity. Under physiological conditions, little *in vivo* evidence of GrK-mediated extracellular cleavage of substrates exist. Yet, accumulating *in vitro* evidence points to the roles of GrK in endothelial activation and the induction of a pro-inflammatory cytokine response.

### Endothelial Activation

Following an infection the endothelium undergoes changes, defined by the expression of cell-surface adhesion and endothelial leukocyte adhesion molecules, to participate in the inflammatory response - a process known as endothelial activation. GrK activates the endothelium through modulation of transmembrane receptors. Through cleavage, GrK activates a member of the protease activated receptor (PAR) family, PAR-1 *in vitro*, which is considered an important activator of endothelium (39, 57). PAR-1 is activated by cleavage of its N-terminal by proteases. GrK-mediated endothelial activation is abrogated by interference of the neutralizing antibody for PAR-1, ATAP-2, suggesting GrK-mediated endothelial activation is dependent on cleavage and activation of PAR-1 (39, 40, 57). Differential responses from the endothelium are induced depending on the cleavage site of the receptor, conferred by the co-receptor and protease utilized (e.g. thrombin cleavage of PAR-1 induces a pro-inflammatory response in cells, whereas APC induces an anti-inflammatory response) (58).

GrK administration is not cytotoxic to endothelial cells and leads to PAR-1-dependent increased expression of the adhesion molecules intercellular adhesion molecule-1 (ICAM-1), E-selectin and vascular cell adhesion molecule-1 (VCAM-1) *via* mitogen-activated protein kinase (MAPK) p38 phosphorylation (39), which facilitate the recruitment and adhesion of circulating leukocytes (59) (**Figure 1**). Additionally, *in vitro* studies indicate that endothelial activation is enhanced upon GrK administration to tumor necrosis factor α (TNF-α)-pretreated human umbilical vein endothelial cells (HUVECs), suggesting GrK augments TNF-α-mediated endothelial activation (39). In addition to expression of adhesion molecules, GrK-mediated PAR-1 activation leads to production and secretion of cytokines, thereby promoting inflammation (39).

### Pro-Inflammatory Cytokine Response

Several granzymes have been reported to modulate the pro-inflammatory cytokine response [e.g. GrA (200-600 nM), GrB (25-100 nM)] (8, 41, 51). In addition to the prior mentioned intracellular modulation of mGrK resulting in release of inflammatory cytokine IL-1β, extracellular human GrK has been reported to cause a pro-inflammatory cytokine response *in vitro*. This response is observed in monocytes, human lung fibroblasts, HUVECs, human keratinocytes and skin fibroblasts (39, 40, 56, 60). The release of pro-inflammatory cytokines from these cells is dependent on PAR-1 activation and downstream extracellular signal-regulated kinase 1/2 (ERK1/2) and MAPK p38 phosphorylation and independent on nuclear factor-kB (NF-kB) (39, 56, 60) (**Figure 1**). HUVECs, human keratinocytes, skin fibroblasts and pulmonary fibroblasts shown an enhanced expression of IL-6 upon GrK administration, which may influence inflammation through leukocyte differentiation (39, 40, 56). Likewise, monocyte chemoattractant protein 1 (MCP-1) secretion is enhanced in HUVECs and pulmonary fibroblasts when treated with GrK, stimulating inflammation by attraction of leukocytes (39, 40). Lastly, GrK treatment induces enhanced IL-8 release from human lung fibroblasts (39, 40). These cytokines all promote inflammation by development, recruitment and activation of immune cells (61, 62). Therefore, next to intracellular functioning, the potential of GrK to activate the endothelium and induce a pro-inflammatory cytokine response through PAR-1 point towards a role for GrK in inflammatory diseases. GrK stimulates a pro-inflammatory cytokine response in both human and mice, suggesting an essential role of GrK in the production of cytokines and control of inflammation during evolution. This is further supported by the notion that the GZMA/K locus exists in representative species tracing back to cartilaginous fish (63).

## GrK in Disease

Similar to other proteases, intracellular and extracellular granzyme levels are increased during several diseases, indicating the significance of progressive granzyme research. Emerging evidence on the pro-inflammatory potential of granzymes further underlines the suggested role of granzymes in disease (8). As this is a newly emerging field, most research consists of *in vitro* studies providing insight into the role of granzymes in disease, including GrK. Human research demonstrates free GrK levels are elevated in several viral and bacterial infections, sepsis (14, 60), burn wounds (56) and airway infections including allergic asthma and acute bronchopneumonia (64) (**Table 2**).

**Table 2:** Intracellular and extracellular GrK in human disease.

Disease Status	Extra- or intracellular	Description	Reference
**Viral infection**			
Influenza A virus	Intracellular	GrK cleaves importin 1α or β *in vitro*, inhibiting viral replication of influenza A.	(38)
Dengue virus	Extracellular	Soluble GrK levels are elevated, suggesting an anti-viral role of GrK *in vivo*.	(23)
Cytomegalovirus	Extracellular	Soluble GrK levels are elevated, suggesting an anti-viral role of GrK *in vivo*.	(23)
**Bacterial infection**			
*Pseudomonas aeruginosa*	Extracellular	GrK synthesis occurs after 24h incubation of whole blood with *P. aeruginosa.*	(65)
Sepsis	Extracellular	Free GrK (monomer) is only found in septic patients, compared to the inactive (multimer) form in healthy controls.	(14)
Experimental endotoxemia	Extracellular	GrK levels are elevated upon LPS injection.	(65)
**Lung disease**			
Airway inflammation (Allergic asthma & Bronchopneumonia)	Extracellular	GrK levels are elevated compared to healthy controls, leading to CCL3 release and recruitment of T cells to the site of inflammation.	(62)
**Other**			
Thermal injury	Extracellular/ intracellular	GrK impairs wound healing in mice by promotion of inflammation and inhibiting epithelialization.	(56)

GrK, granzyme K; CCL3 or MIP-1-alpha, Macrophage inflammatory protein-1α.

### Viral Infections

Cytomegalovirus (CMV) is a herpesvirus affecting primates, including humans (66). As a non-life threatening virus, most infected people do not notice CMV, whereas in immunocompromised patients (e.g. AIDS patients) a CMV infection causes symptoms including diarrhea and fever (66). In human plasma, GrK levels are elevated in patients suffering from CMV infection (23). Moreover, GrK levels are elevated in plasma samples from Dengue fever patients compared to healthy controls (23). Plasma-derived cytotoxic T lymphocytes of mice infected with Chikungunya virus have increased expression of GrK (67). Furthermore, GrK^-/-^ mice have reduced foot swelling compared to GrK^+/+^ mice, suggesting a pro-inflammatory role for GrK (67). The enhanced production and secretion of GrK in these diseases underlines the potential of GrK to play a role in the immune defense against viral infections. Whether GrK modulates the response to these viral infections and the molecular mechanisms involved remain to be explored.

Influenza A is a recurring virus characterized by fever and coughing which causes epidemics in birds and mammals (68). When challenged with influenza A, WT mice display long-term expression of GrK by antigen-specific CTLs (69). Moreover, GrK was detected at high frequencies in CD8+ T cells derived from GrA^-/-^/GrB^-/-^ mice after challenge with influenza A (70). Influenza A is a negative-strand RNA virus replicated by RNA polymerase in the host cell nucleus through recognition of the nucleoprotein (NP) and viral RNA complex (38). Prior to complex formation, RNA polymerase and NP are transported from the cytoplasm to the nucleus by binding to importin 1α or β (38). Human GrK associates to importin 1α and cleaves importin 1α and β following incubation with K562 cell lysates. Therefore, GrK may inhibit viral replication by cleaving importin 1α or β, preventing NP/viral RNA complex formation and preventing RNA polymerase recruitment *in vitro* (38). The finding GrK may inhibit viral replication by its proteolytic capability has yet to be confirmed for humans and mice *in vivo*.

Furthermore, GrK possibly aids in the clearance of LCMV infection in mice (17). LCMV is a rodent virus which is a widely accepted model to study viral infections. GrA and GrB knockout mice models suggest that GrA and GrB are not imperative for LCMV clearance in mice, in contrast to perforin (17). This, together with the finding that GrK is expressed *ex vivo* by LCMV-infected mouse-derived CD8+ T cells, led to the hypothesis that GrK might control LCMV clearance in mice (17). LCMV-derived CD8+ T cells expressing GrK but not GrA and GrB are not cytotoxic (17). Accordingly, administration of recombinant GrK is not cytotoxic to EL4 cells *in vitro* (17). However, recombinant GrK induces production of mature proinflammatory IL-1β in pre-activated PEMØs (macrophages) (17). Similarly, LCMV-derived immune cells and GrA^-/-^/GrB^-/-^ CD8+ cells induce release of IL-1β in LPS-primed PEMØs in the presence of gp33, a LCMV-immunogenic peptide (17). IL-1β may be an important mediator in LCMV infections since mice treated with IL-Ra, a IL-1β receptor antagonist, fail to clear the LCMV infection (17). Combined, these results suggest that clearance of LCMV infection is (at least partly) dependent on GrK-mediated non-cytotoxic mechanisms (17). However, the suggestion that GrK is a key player in LCMV clearance is challenged by an *in vivo* study on GrK^-/-^ mice, which show no impaired LCMV clearance upon intraperitoneal injection with LCMV compared to WT mice (48). Moreover, elimination of Ectromelia virus (ECTV) is not impaired in GrK^-/-^ mice. This suggests GrK does not play an essential role in the anti-LCMV or anti-ECTV immunity (48).

### Bacterial Infections

Intake of pathogenic bacteria derives from different kinds of sources such as food or water consumption, air, living vectors, or indirect contact. Infections with gram-negative or gram-positive bacteria activate a variety of molecular pathways and symptoms observed in an infected individual (e.g. fever, inflammation, swelling) dependent on the bacterial and cell wall characteristics (e.g lipopolysaccharide (LPS) from the outer cell membrane of gram negative bacteria). More serious bacterial infections can result in septic shock, characterized by organ failure and ultimately lead to death if left untreated.

In sepsis and human experimental endotoxemia, a model for systemic infection, levels of soluble GrK, GrA and GrB, are elevated (14, 60, 71). The elevation of GrK in sepsis is accompanied by a reduced expression of IαIp, the natural inhibitor of GrK, which indicates an increase in the activity of GrK (inverse correlation) (32, 65, 72). Subsequent research showed GrK release in whole blood cultures is restricted to *P. aeruginosa*, a gram-negative bacterium (73). This suggests that the gram-negative bacterial cell wall, in particular LPS, plays a pivotal role in triggering GrK secretion.

*In vitro* research confirmed this hypothesis. GrK, and its catalytically inactive mutant, GrK-SA, have been shown to bind and modulate LPS – suggesting that LPS binding is independent from GrK’s catalytic activity (60). LPS consists of a lipid that inserts the molecule in the membrane, a core peptide and the O-antigen. Studies focusing on recombinant LPS revealed GrK potentiates LPS by binding to the O-antigen, the outermost part of LPS (60). Recombinant LPS molecules exist in plasma as micelles, with protruding O-antigen faces towards the extracellular space (60). GrK facilitates both removal of individual LPS molecules from micelles by binding to its O-antigen, and their transfer to CD14. LPS and CD14 form a complex that binds to toll-like receptor 4 (TLR4) on the cell membrane, leading to an inflammatory cytokine response (60). Specifically, it was shown *in vitro* that this leads to TNF-α release from human primary monocytes (60).

### Airway Inflammation

In lung diseases such as chronic obstructive pulmonary disease (COPD) (a progressive inflammatory lung disorder characterized by shortness of breath and coughing), hypersensitivity pneumonitis (rare immune system disorder affecting resulting in hypersensitivity to inhaled dust) and allergic asthma (resulting from exposure to allergens e.g. pollen), roles for GrA and GrB have been reported. Less is known about the endobronchial expression and release of GrK in lung disease. In a study involving non-smoking and smoking subjects with or without asthma, bronchopneumonia or COPD, bronchoalveolar lavage fluid (BALF) of acute bronchopneumonia patients showed a 18-fold increase of GrK compared to healthy controls (64). Similarly, in allergic asthma patients an elevation in soluble GrK levels as well as GrK expressing CD8^+^ T cells in BALF could be observed upon allergen challenge (24 and 72 hour after exposure) (64). Recruitment of GrK expressing CD8^+^ T cells might be dependent on chemokine c-c motif ligand (CCL)3, a chemokine which is elevated in the BALF of asthma patients following allergen challenge (64). Further, *in vitro* studies demonstrate that extracellular GrK induces cytokine secretion of IL-6, IL-8 (CXCL8) and monocyte chemotactic protein-1 (MCP-1)/CCL2 and proliferation of human lung fibroblasts through a PAR-1 dependent mechanism (40). This suggests GrK may play a role in airway remodeling by augmenting inflammation (40, 64).

### Inflammaging

Aging and age-related illnesses share similar mechanistic processes converging on inflammation (74). Affecting the immune system, aging results in a chronic low-grade inflammation (inflammaging) that contributes to the pathogenesis of age-related illnesses. Interestingly, a recent study of Mogilenko et al., 2021 identified a subset of clonal GrK^+^ CD8^+^ T cells as conserved hallmark of inflammaging in both mice and humans. In mice of advance age nearly half of all circulatory CD8^+^ T cells acquire an age-associated GrK^+^ CD8^+^ T phenotype indicating the potential impact on aging physiology through GrK secretion. Furthermore, in a healthy human cohort clonal GrK^+^ CD8^+^ T cells increased with age. Like in mice, the cells display elevated EOMES expression levels and a distinct epigenetic landscape. Together, the results suggest that GrK^+^ CD8^+^ T cells as well as GrK itself can be potential targets to address age-associated immune dysfunction (75).

### Thermal Injury

Healing of thermal injury and burn wounds is often accompanied by inflammation, leading to painful contractures and excessive scarring (76). In humans, levels of extracellular GrK are elevated in acute burn tissues following thermal injury compared to normal tissue (day 2-30 after injury). This is predominantly observed in macrophages (56). Furthermore, after GrK^-/-^ mice were subjected to thermal injury (grade 2), they showed improved matrix organization, wound closure, dermal maturation, enhanced re-epithelization and tensile strength in comparison with WT mice. The GrK^-/-^ mice also exhibit reduced expression of pro-inflammatory IL-6, IL-1β, MCP-1, ICAM-1 and VCAM-1 (3 days after injury), suggesting a delayed pro-inflammatory response (56). Accordingly, a reduced infiltration of M1 macrophages was observed in burn injury of GrK^-/-^ mice compared to WT mice (56). Cell migration of keratinocytes significantly decreased and impaired re-epithelialization was observed in GrK+ mice compared with GrK^-/-^ mice (56). *In vitro* exposure to GrK in keratinocytes, but not skin fibroblasts, demonstrated impaired wound healing (56). Combined, GrK may delay thermal injury-related wound healing by the promotion of pro-inflammatory cytokine expression and impaired re-epithelization. This potential GrK role is reminiscent of GrB, which delays skin wound healing in mice through activation of the pro-inflammatory cytokine response and degradation of extracellular matrix components (77). Consequently, therapeutic targeting of GrK may relieve disease burden given the potential roles of GrK in infections (viral or bacterial), airway inflammation and thermal injury.

## GrK as a Therapeutic Target

Since granzymes are associated with several diseases and appear extracellularly, they are considered promising therapeutic targets (8). Several fusion proteins of GrB against solid tumors are in development as therapeutic agents, such as GrB conjugated to VEGF or TNF-α (78). Similarly, inhibition and administration of GrK could provide novel ways to overcome disease.

Inhibition of GrK could be beneficial for diseases in which elevated GrK is associated with unfavorable disease outcomes. For example, GrK inhibition could reduce the release of pro-inflammatory cytokines in allergic asthma. Over the last 25 years, several inhibitors for GrK have been described including physiological inhibitors, and specific and nonspecific synthetic inhibitors (32). Physiological nonspecific inhibitors of GrK include antithrombin III (ATIII) and α-macroglobulin (α-2M) (32), mainly inhibiting thrombin, plasmin, cathepsin G and blood coagulation factors (79, 80). The GrK inhibitory effect of these compounds is elevated when combined with heparin (27, 32). However, only high, non-physiological concentrations of ATIII and α-2M reduce the catalytic ability of GrK (32). Up to date, the only identified specific physiological GrK inhibitor in human plasma is IαIp (32). IαIp inhibits GrK dose-dependently as well as inhibiting cytokine production (32, 39, 81). IαIp contains various chains, one of which is bikunin. Bikunin also circulates as free form in plasma inhibiting GrK at its S1 pocket (32). Administration of (human) IαIp in mice, rabbit and rat models ameliorates survival in LPS or bacteria-induced sepsis (82–85). Considering that GrK and IαIp levels in plasma of sepsis patients are elevated, these could be used as molecular targets or treatment for sepsis patients (72). Synthetic GrK specific inhibitors include 3,3-diphenylproponyl-Pro-(4-AmPhGly)P(OPh)2 and D-Phe-Pro-Arg-chloromethyl ketone (30, 86, 87). Other general synthetic protease inhibitors (e.g. trypsin inhibitors benzamidine and aprotinin) also inhibit GrK, as well as GrA activity (32, 87, 88).

In infections with gram-negative bacteria, the unfavorable pro-inflammatory cytokine response cannot be decreased by an inhibitor targeting GrKs active site, as GrK induces the pro-inflammatory cytokine response by binding to LPS and transferring LPS to CD14. Monoclonal antibodies that interfere in the interaction between GrK and LPS are proposed as potent mediators (60). This approach could also be valuable in other diseases in which GrK plays a pathogenic role independent of its catalytic activity. To date however, no monoclonal antibodies to target GrK therapeutically have been reported. Moreover, targeting proteases that activate PAR-1 to stimulate the inflammatory response might thus provide an efficient therapeutic strategy as PAR-1 adopts a dual role in disease, both protective and pro-inflammatory (39).

Furthermore, GrK could be targeted by attenuating gene expression. Gene expression can be influenced by small interfering RNAs (siRNAs) or microRNAs (miRNAs). Targeted siRNAs are already in use to knockdown granzyme expression, for example in granzyme C research (89). Next to siRNAs, miRNAs interfere in the post-translational modification of newly synthesized granzymes, including GrK. Recently, miRNA-145 has been reported to be valuable in myocardial ischemia/reperfusion (I/R) injury mouse models by regulating the expression of GrK. In this study, the protective role of miRNA-145 against I/R injury by regulating the expression of GrK with the treatment of anesthetic sevoflurane was investigated. In ischemia, miRNA-145 levels are decreased, whilst mGrK levels are upregulated. As GrK is a potential target gene of miRNA-145, miRNA-145 could inhibit GrK expression. Upon treatment with sevoflurane, miRNA-145 levels are upregulated and GrK expression is reduced – relieving I/R injury (90). Interestingly, miRNA-145 significantly elevates functioning of the left ventricle and decreases the myocardial infarct size suggesting that downregulation of GrK and upregulation of miRNA-145 may be protective of I/R injury (90).

GrK administration could be beneficial for diseases in which GrK expression is associated with favorable disease outcomes (e.g. recovery). This includes for example influenza A infection. Administration of exogenous granzymes has been studied extensively for human GrB *in vitro* and in mice using recombinant GrB coupled to the Lewis Y-binding antibody dsFv-B3 or using an anti-HER2 antibody against HER2 tumors (91, 92). Through this granzyme-antibody construct, GrB can be effectively internalized both *in vivo* and *in vitro* (92). Illustratively, by conjugating GrK to an anti-sialic acid antibody, the influenza A receptor, GrB delivery could be adjusted to GrK.

## Conclusion

After twenty years of GrK research, new roles are emerging complementing GrK’s traditionally described role in cytotoxicity. Most current studies on GrK discussed in this review are either *in vitro* (e.g. cell-culture, binding assays) using human or mouse GrK or *in vivo* animal studies (e.g. GrK^-/-^ mice). Both *in vitro* and *in vivo* studies identified roles in the modulation of inflammation, inhibition of viral replication and LPS potentiation. Furthermore, GrK inhibition and stimulation have been suggested for a variety of disease statuses as therapeutic targets, including inflammatory disease and cancer. Whilst no longer being considered an ‘*orphan granzyme’* at present, much of GrK functioning and molecular mechanisms remain to be discovered.

Targeting GrK therapeutically remains a challenge due to a lack of *in vivo* studies involving GrK in disease and GrKs dual role in pathology (for example in viral replication inhibition and airway inflammation). To meet the latter difficulty, GrK-SA may be used as a substitute for active GrK in therapy. This way, the pathological effect dependent on the active site of GrK should be reduced, thereby minimizing adverse effects of GrK administration. Further research is required to i) explore the controversy around GrKs cytotoxic potential ii) review the inhibitory effect of GrK on influenza A replication *in vivo* and iii) investigate the curative role of GrK(-SA) upon inhibition (with synthetic or physiological inhibitors) or administration in thermal injury and airway inflammation.

## Author Contributions

All authors listed have made a substantial, direct and intellectual contribution to the work, and approved it for publication.

## Funding

KD received funding for het PhD studies from the Gates Cambridge Scholarship (OPP1144).

## Conflict of Interest

The authors declare that the research was conducted in the absence of any commercial or financial relationships that could be construed as a potential conflict of interest.

The reviewer DK declared a past co-authorship with one of the authors NB to the handling editor.
